# Positive deviance/hearth intervention in collaboration between academia and NGOs: a realist evaluation

**DOI:** 10.1186/s12889-024-20632-4

**Published:** 2024-12-28

**Authors:** Nuzulul Kusuma Putri, Leonika Pramudya Wardhani, Ernawaty

**Affiliations:** 1 Health Policy and Administration Department, Faculty of Public Health Universitas Airlangga, Surabaya, 60115 Indonesia; 2 International Health and Sustainable Development Department, Tulane University Celia Scott Weatherhead School of Public Health and Tropical Medicine, New Orleans, 70112 United States; 3 The Airlangga Centre for Health Policy Research Group, Surabaya, 60115 Indonesia; 4 Research Group of Health Policy and Administration, Universitas Airlangga, Surabaya, 60115 Indonesia

**Keywords:** Child health, Higher education policy, Nutrition, Public-private partnership, Positive deviance

## Abstract

Numerous NGOs and donors from high-income countries (HICs) offer diverse funding to assist LMICs. A collaborative effort in nutrition programs in urban communities was conducted by an international NGO with a local university, representing the NGO’s first mode of partnership with academia. This study used realist evaluation to understand how and why Positive Deviance/Hearth intervention conducted by NGOs collaborated with university work or failed to work in urban population. We uncovered the underlying mechanisms that lead to certain outcomes in different situations of academia-NGO partnership. The initial program theory was formulated after discussions with main program officers from both NGO and academia. We conducted stakeholder interviews and FGD, along with validation and reviewing secondary data. The initial program theory underwent testing and refinement through a series of Focus Group Discussions (FGDs) involving program officers and local government health offices. We adhered to the RAMESES II reporting standards for realist evaluations when presenting the findings. Five Context Mechanism-Outcome (CMO) configurations are identified from three program theories. Our findings illustrate the importance of considering the local context of the population when implementing standardized international NGO interventions. All the CMOs indicate that at the very least, collaborative programs between academia and NGOs should involve more stakeholder involvement and build clearer expectations between stakeholders.

**Supplementary Information:**

The online version contains supplementary material available at 10.1186/s12889-024-20632-4.

## Introduction

Low- and middle-income countries (LMICs) are often the focus of global health interventions. Numerous NGOs and donors from high-income countries (HICs) offer diverse funding to assist LMICs. Nevertheless, the improvements in some assisted health indicators are not supported by a prioritization framework, such as enhancing local health systems, which jeopardizes their sustainability and justice [1]. There are indications that a clear, proactive mechanism is lacking to ensure greater equity in funding and interventions [2]. Nutrition interventions have been long time critiqued as solely conducted based on the theory that the poor only need to be fed to escape from their poverty and tend to stereotype poverty as only a hunger problem [3].

Every child has the right to nutritious food. However, due to inadequate nutritious food, at least 22.3% of children under five experience stunting (low height for age), and 6.8% experience wasting (low weight for height) [4]. Of that number, 52% of stunting and 70% of wasting occurred in Asia countries. While in Southeast Asia, even though hunger is less alarming than in other regions worldwide, 27.4% of its children under five are stunted, 8.2% are wasted, 7.5% are overweight, and one among two children experience hidden hunger due to micronutrient deficiencies [5].

Toddler nutrition status has become a big issue in Indonesia. According to the World Health Organization (WHO), Indonesia’s stunting prevalence is 30.8% and wasting is 10.2% in 2022. Other United Nations Children’s Fund (UNICEF) data reported that it has persistently remained high over the past decade. Therefore, the Indonesian president declared that the convergence, coordination, and consolidation of programs across various levels of government and sectors are needed. Indonesia practices specific and sensitive interventions to reduce its stunting prevalence, which involves multisectoral interventions addressing the complex social determinants of stunting. However, these multisectoral interventions encompassing health, education, sanitation, and nutrition components have not significantly reduced the prevalence of stunting [6].

The Positive Deviance/Hearth (PD/Hearth) intervention aims to rehabilitate malnourished toddlers by utilizing positive practices observed in mothers from economically disadvantaged families with well-nourished children. The concept of PD/Hearth has been implemented worldwide and in various parts of the world and has proven successful not only in improving toddler feeding practices by caregivers during the rehabilitation phase of underweight but also in preventing stunting in healthy toddlers [7, 8]. PD/Hearth also accelerates community awareness about nutrition issues, collective decision-making, motivation for change, advocacy, and adopting new behaviors to prevent toddler malnutrition [9]. PD/Hearth conducted in rural Zambia showed that this intervention effectively reduced the prevalence of underweight [10].

In 2022, the initial implementation of the PD/H intervention was introduced in an urban community in Indonesia within one of the mentoring areas overseen by an international Non-Governmental Organization (NGO). The NGO partnered with a national university in the city where the targeted areas were located to initiate this pilot project. This collaborative effort represents the NGO’s first mode of partnership with academia, which they initiated to facilitate the operationalization of their program. The rationale for conducting this realist evaluation is to inform what should be considered when implementing nutrition interventions led by non-government organizations in the local setting. Evaluating how this intervention works in an international NGO collaborating with a local university is important to guide considerations for shaping partnerships in decolonizing global health for sustainable community development initiatives.

This study used realist evaluation to understand how and why Positive Deviance/Hearth intervention conducted by NGOs collaborated with local university work or failed to work in particular contexts.

## Method

This research employed the realist evaluation methodology, which delves into the interplay between the policy context, the implementation mechanism, and the anticipated policy outcomes. A fundamental principle guiding the use of realist evaluation in implementing nutrition interventions conducted in collaboration with NGOs and academia is understanding the mechanism and outcome of how this partnership functions for the local context of the population and different stakeholders, with their specific barriers and triggers benefiting this partnership. Therefore, realist evaluation is particularly suitable for examining how academic-NGO partnership functions in a program implemented across diverse stakeholders, all operating within complex interactions. Throughout this article, we adhere to the RAMESES II reporting standards designed for this methodology.

To guide this evaluation, we formulated the following research questions: (1) Based on the context in urban communities, how did PD/H intervention by non-governmental organizations impact local communities? (2) What mechanisms underlying NGO collaboration with local universities explain the effects of these interventions?

### Setting

An international NGO focusing on children’s welfare has worked in Indonesia for over eight years. The area programs in Indonesia are diverse, and they are for urban and rural communities. In May 2022, this NGO initiated a partnership with the School of Public Health of a national university to carry out various interventions for the community in their designated area in one of the sub-districts in urban Surabaya. The NGO has been supporting this Sub-district, including two target neighborhoods for PD/H, with the PD/H program and other community empowerment programs around sanitation and child education.

The partnership contract with the university includes all of the programs with funding from the NGO according to their strategic plan. The university is given access to intervention design but must adhere to the NGO guidelines and perform to achieve the NGO’s targets. One of the interventions included in this partnership is the PD/H program. The first cohort of this intervention began in July 2022 and concluded in September 2022. In 2023, the NGO resumed its second PD/H intervention in the same neighborhoods with different children as the target population. To implement PD/H, both recruited student volunteers. The NGO involved their community volunteers who had been long before partnering with the university. For this partnership, the NGO appointed one of its employees as the PD/H coordinator, and the university appointed one of its assistant professors as the partnership coordinator.

**Fig. 1:**
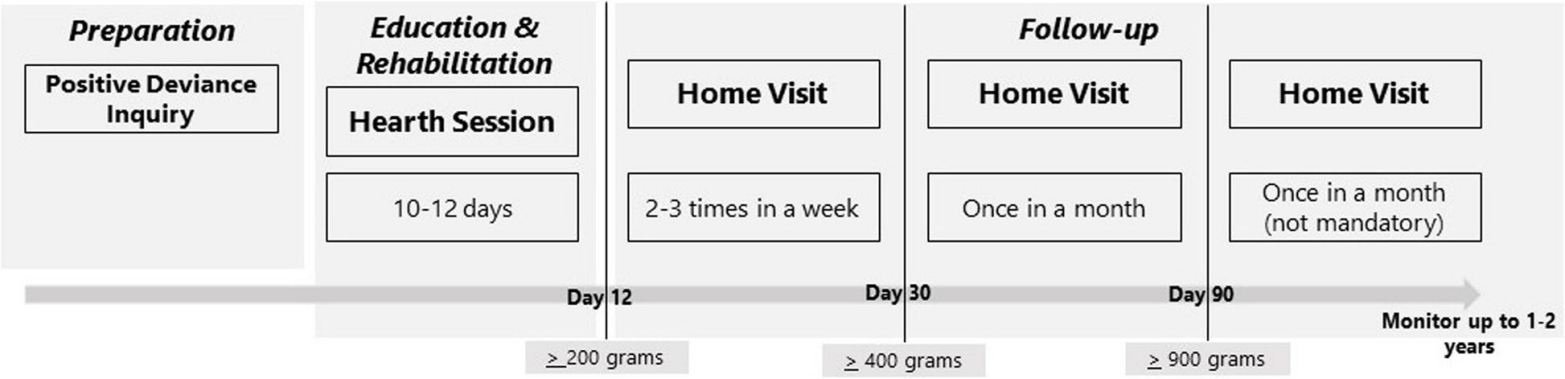
PD/H intervention

The PD/H consists of three stages: preparation, education and rehabilitation, and follow-up (please see Fig. 1 for the detailed intervention). The PD/H coordinators are trained to mobilize the community and train the volunteers. During the first stage, Positive Deviance Inquiry (PDI) is conducted to identify local practices that promote good nutrition. Based on these identified local practices, the program develops Hearth menus utilizing Positive Deviance (PD) foods and other nutrient-rich foods that are locally available and affordable. Children and their caregivers are then invited to participate in 10–12 days of Hearth sessions, during which they prepare and consume Hearth menus while discussing the positive deviant practices. At the end of each stage, the target of weight gain must be achieved. The continuity of this feeding program is maintained through follow-up visits by volunteers, who assess progress and reinforce positive deviant practices in the homes of Hearth graduates. Community stakeholders should be identified and coached at each stage to facilitate and oversee the process.

### Data collection methods, recruitment process, and sampling strategy

#### Phase 1: identifying program theories

LPW (the second author) interviewed the NGO program coordinators and academia coordinators. For the first phase, these participants were sampled purposively based on their role in the program. Based on recommendations from the NGO facilitators, two subsequent interviews were conducted, including a health cadre (community volunteers from the sub-districts) and a student volunteer. The interviews were used to prepare conjectured Context-Mechanism-Outcomes (cCMO), which will be tested in the second phase. cCMO is the hypothesis on the relationship between contextual factors, intervention mechanisms, and expected outcomes. During this phase, researchers also identified various secondary documents related to policies, programs, and activities of PD/hearth that had been conducted in the neighborhoods to validate the cCMOs. At the end of the interview, the researcher asked the participants for recommendations on who should be interviewed next based on certain aspects noted by the researcher during the previous interviews.

#### Phase 2: testing and refining program theories

The cCMOs generated from phase 1 were tested by conducting Focus Group Discussions (FGD) with PD/hearth stakeholders. Findings from the second-phase FGDs were used to enhance the cCMOs generated in phase 1. In this phase, participants from the first phase were involved, and two additional cadres, two student volunteers, and nutrition officers from the Public Health Center (Puskesmas) who were involved in the program were included. These additional names were obtained based on recommendations from each interview to accommodate the snowball sampling process.

Appendix 1 lists the participants for phase 1 and phase 2. The questions used for these phases are presented in Appendix 2.

### Data management

All audio recordings were stored on a personal password-protected laptop, accessible only to individual researchers. The research team transcribed interviews verbatim. The first phase of interviews resulted in 14 pages of single-space transcription with 40 min of interviews for each participant. For the second phase, we got seven pages of single-space transcription from an hour of FGD. All transcripts were uploaded into NVivo 12 for coding and analysis, a co-sharing mode between researchers (NKP, LPW, ERN).

### Data analysis

Thematic analysis was employed to construct the cCMO (Context-Mechanism-Outcome) framework. Deductive analysis was used to code the findings of the Phase 2 Focus Group Discussions (FGDs) in alignment with the cCMO generated in Phase 1. Additionally, inductive analysis was conducted to identify themes not covered in the cCMO and to explore the connections between the assembled CMOs. The coding book (see Appendix 3) is structured by researchers before data collection based on the realist evaluation components. The researchers created the code independently by referring to the book, given the freedom to develop or modify it according to the understanding they gained during the analysis process.

### Validity strategy

Two researchers (NKP and LPW) did the initial coding of each transcript. When NKP and LPW met any differences in the individual coding or creating new code in the coding book (Appendix 3), a discussion was hosted to achieve a consensus involving ERN as the third researcher. Thematic saturation was used to triangulate the data. NKP, LPW, and ERN were discussed to ensure thematic saturation, where no new themes or significant additional information emerged in the data analysis.

## Results

Based on program records, out of a total of 37 children who participated in PD/H, 67.6% of them had normal nutritional status based on weight-for-age on the first day of this intervention. Weight gain on day 10/12 appeared promising, with very few children experiencing weight loss. However, on day 30 or 1 month after the hearth session, there was a decrease in the percentage of children with normal weight-for-age to 48.6%. On day 90, the percentage decreased to 56.8% of children with normal nutritional status based on weight-for-age. Poor monitoring and evaluation were the most plausible reasons for the drop in progress. On day 60, there was a rapid increase in the missing data on the toddler’s condition. Their weight gain data could not be monitored during that period.

### Phase 1: identifying program theories

The conjectured Context-Mechanism-Outcomes (cCMOs) were derived from an inductive analysis of the interview findings in the study’s first phase. We identified three primary theories operating within different contexts and mechanisms influencing the program outcomes.

The first theory revolves around the involvement of parents during the program, which is closely linked to the urban setting in which the PD/H program was observed. In this urban environment, where most parents were employed, the active participation of parents was considered pivotal. Secondary data regarding the presence of parents during PD/H activities revealed a lack of parent commitment, with parents frequently arriving late for the heart session or skipping them altogether.

Another theory pertains to the collaborative framework involving academia and an NGO. Within this framework, students represent a significant resource for academia in implementing the program, while the NGO already has community members with long-standing affiliations. However, when the partnership took place, these groups brought different backgrounds and experiences to the table as they adapted to working together. In the context of this partnership, our interviewees predominantly highlighted the absence of standardized qualifications and the varying workloads among these diverse human resources components.

The third theory that emerged concerns the PD/H program’s nature as a community empowerment intervention. The participants regarded this theory, which centers around the characteristics of the community and the program initiator, as a significant context with distinct mechanisms influencing the program outcomes.

We built different cCMOs for each theory. Table 1 presents the cCMOs. Subsequently, these conjectured Context-Mechanism-Outcomes (cCMOs) were submitted for examination in the subsequent phase of the study.

**Table 1:** Conjectured context-mechanism-outcomes (cCMOs) at the end of phase 1

Theory	Conjectured context mechanism outcome configuration
involvement of parents during the program	cCMO 1 When working parents cannot escort or accompany their children during hearth sessions (C), it necessitates parental commitment and involvement in participating in the program (M) to support the swift rehabilitation of children experiencing wasting and underweight (O).
collaborative framework involving academia and an NGO	cCMO 2 The program implementers, who come from diverse backgrounds (C), should be supported by establishing standardized qualifications and a proportional workload (M) to achieve the rapid rehabilitation of children experiencing wasting and underweight and to prevent wasting and underweight in the future (O).
	cCMO 3 The characteristics of toddlers in urban areas, where there is a high incidence of infectious diseases (C), necessitate the implementation of proper sanitation management and preventive measures through immunization or vaccination at healthcare facilities (M). This approach aims to prevent wasting and underweight in the future and to maintain recovery outcomes (O).
the nature of the PD/H program as a community empowerment intervention	cCMO 4 When replicating a community empowerment program in a different location using the same implementation guidelines (C), the application should be adjusted to the specific conditions of the target community (M). This adaptation helps in maintaining recovery outcomes in the target area and facilitates behavioral change in that community (O).
	cCMO 5 When replicating a community empowerment program in a different location using the same implementation guidelines (C), it is essential to establish consistency in the procedures performed by program implementers (M). This consistency is crucial for maintaining the program’s recovery outcomes (O).
	cCMO 6 Programs initiated by non-governmental organizations (C) require support in the form of commitment and engagement from various sectors, such as health centers, neighborhoods, and local community organizations (M). This support is necessary to sustain the recovery outcomes (O).

### Phase 2: testing and refining program theories

We conducted a Focus Group Discussion (FGD) with diverse participants to compare and contrast how PD/H was implemented through a partnership between academia and an NGO. Based on the FGD results, the six cCMOs that were tested were divided into five refined CMOs. CMO 4 combines cCMO 4 and 5, while cCMO 6 becomes CMO 5.

#### CMO 1—When working parents cannot escort or accompany their children during heart sessions (C), parental commitment and involvement in participating in the program (M) is necessary to support the swift rehabilitation of children experiencing wasting and underweight (O)

This study has identified a distinctive feature of urban areas in executing the Positive Deviance/Hearth program: a substantial portion of parents, particularly caregivers, are employed. The program caters to children aged 6–36 months, with parents playing a pivotal role in their upbringing. Consequently, working parents in urban settings must be highly committed to the program. The extent of their commitment and involvement will be crucial to the program’s effectiveness in swiftly addressing wasting and underweight issues.

*“But it is very difficult in Surabaya because Surabaya is an urban context. Positive Deviance/Hearth is not very effective in urban cases. Behavioral change becomes difficult because both parents work” (Female*,* NGO Facilitator*,* March 2023).*

When considering the commitment and involvement of parents in enrolling their children in the Positive Deviance/Hearth program, it becomes evident through the active participation of mothers in accompanying their children to Hearth sessions and their dedication to attending these sessions consistently for 10–12 days. However, the program’s design has failed to consider the urban implementation context, where most parents are employed. Caregivers eager to attend daily sessions for ten consecutive days find it exceedingly challenging within urban settings.

*“At first*,* many came*,* but then the numbers kept decreasing. Some parents are too lazy to bring their children to Nutrition Pos” (Female*,* program cadre*,* March 2023).*

These statements are further supported by other participants who mention that parents are unwilling to participate in the program.

*“…there may be children with a WAZ score <-1*,* but their parents are not willing to participate in this program. Because this program is voluntary in nature” (Female*,* NGO facilitator*,* March 2023).*

The commitment of mothers to implement the meal plan at home after participating in the program is also considered insufficient by program cadre participants.

*“I think another obstacle is that parents are lazy*,* sister. The meal plan demonstrated during cooking demos is easy and practical*,* sister. It’s easy but seems difficult for parents to practice at home” (Female*,* health cadre*,* March 2023).*

From the perspective of the cadre, the poor commitment of mothers participating in the program is due to their perceptions, which see this PD/H intervention as a short-term project for which they could get financial rewards.

*“Ideally*,* the mother should cook for her child*,* but we had the opposite. Health cadres were asked to cook by mothers. When we refused*,* they asked the student volunteers. They said they are worried about cooking since the kids need breastfeeding*,* pampered*,* or whatever. When we motivated them to the intervention’s benefits*,* they said*,* “Oh Gosh*,* only that?” (Female*,* health cadre*,* March 2023).*

#### CMO 2 – the program implementers, who come from diverse backgrounds (C), should be supported by establishing standardized qualifications and a proportional workload (M) to achieve the rapid rehabilitation of children experiencing wasting and underweight and to prevent wasting and underweight in the future (O)

The program implementers of the Positive Deviance/Hearth program, including facilitators, student volunteers, and cadres, come from diverse backgrounds in age, education, occupation, and experience. These program implementers are organized into teams or groups placed in different neighborhoods. However, there is often inconsistency in how program procedures are carried out, highlighting the need for standardized qualifications regarding skills, knowledge, commitment to procedures, and clear and equitable workload distribution among program implementers. The effectiveness of the program and its outcomes depend on the qualifications of the human resources involved. This study found that student volunteers had not conducted intensive monitoring and that preparations were rushed. As this was the first time the program was implemented, these issues must be addressed before similar programs are implemented.

*“The monitoring process conducted by volunteers was not very intensive. You can see it in the weighing results. For example*,* on the seventh day*,* weight was significantly increased. However*,* when it comes to days 30*,* 60*,* and 90*,* because it was not well-monitored and the behaviors we taught during PDH sessions were not implemented well at home*,* the child’s weight decreased again” (Female*,* NGO Facilitator*,* March 2023).*

Standardizing qualifications, such as the number of program implementers directly involved, should be carefully considered for optimal results. Otherwise, things may not run smoothly from planning to implementation, monitoring, and evaluation because one person is taking on multiple roles, causing a lack of focus.

*“The limitation of human resources is because few staff members have to handle and plan programs across different fields. This leads to a workload bottleneck*,* resulting in poor planning” (Female*,* academia partnership coordinator*,* March 2023).*

There are still noticeable gaps between different parties within the program implementers’ teams. Due to their backgrounds, one party is considered more knowledgeable about the program than the other, which can create an imbalance in workload.

*“Here*,* as volunteers*,* we help where cadres still need our assistance. Nevertheless*,* most of the tasks are delegated to us*,* the student volunteers. So*,* the cadres tend to follow along. This creates an uneven workload” (Female*,* student volunteer*,* March 2023).*

Workload issues also arise due to a lack of careful workload planning.

*“Yesterday*,* three students and three cadres were handling around 12–15 children*,* which was insufficient. So*,* there might need to be a strategy to calculate the appropriate workload for Nutrition Pos volunteers” (Female*,* NGO Facilitator*,* March 2023).*

Student volunteers’ and cadres’ knowledge of Positive Deviance/Hearth is still not comprehensive, and there are differences in perceptions or concepts related to Positive Deviance/Hearth and program objectives.

*“The challenge is to align the concepts. Here*,* it is not just Nutrition Post (Hearth Session). Nutrition Post involves cooking*,* as mentioned earlier. However*,* here*,* there is a PDH approach. Moreover*,* volunteers carry out this approach. We find it difficult to transfer this knowledge to volunteers and ensure that they can conduct positive deviance investigations in the field according to the material” (Female*,* academia partnership coordinator*,* March 2023).*

#### CMO 3 – the characteristics of toddlers in urban areas, where there is a high incidence of infectious diseases (C), necessitate implementing proper sanitation management and preventive measures through immunization or vaccination at healthcare facilities (M). This approach aims to prevent future wasting and underweight and maintain recovery outcomes (O)

According to the program record, the number of children with improved weight from all the participants consistently mentioned that the weight loss in toddlers who had initially gained weight was due to infectious diseases. Respondents stated that the children’s weight had improved since attending Hearth sessions, but their weight decreased again after monitoring. This was attributed to poor environmental sanitation, weak children’s immunity, and unpredictable weather. Children who contract infectious diseases will hinder the program’s intended outcomes, preventing future wasting and being underweight. Recovery outcomes also cannot be consistently maintained if the child suffers from illnesses like coughs, colds, and fever, as the child becomes fussy and loses appetite.

*“At that time*,* during the implementation of PDH*,* we found that quite a few of them had infectious diseases. Moreover*,* these infectious diseases are the main reason these children did not gain weight” (Female*,* NGO Facilitator*,* March 2023).*

Toddlers require efforts to boost their immunity. Through one of the health empowerment programs coordinated by the Public Health Center (Puskesmas), Posyandu (integrated health post), toddlers are regularly weighed, and their development is checked.

*“If you look at it from the toddler’s condition*,* it’s more about illness. Because there were a lot of them who got sick with coughs*,* colds*,* etc. So*,* to prevent this from happening again*,* it’s recommended to give immunizations/vaccines/vitamins/deworming before the Hearth session begins. Furthermore*,* we can coordinate with the Puskesmas since we do not have the budget for that” (Female*,* academia partnership Facilitator*,* March 2023).*

This condition made the implementers rethink how effective the PD/H intervention is without any modification according to the urban context. It is also related to CMO 4, which encourages any adaptation to the condition of the community.

*“There is a need to evaluate whether the PD/H intervention is effective enough to be carried out in urban settings where not only the parents are working but also its complexity of sanitation problems which create high infectious diseases. Let’s see whether we need to modify PD/H or just run that based on the standard guideline” (Female*,* NGO Facilitator*,* March 2023).*

#### CMO 4—When replicating community empowerment programs in different locations using the same implementation guidelines (C), their application must be adapted to the target community’s culture (M) to build multi-sectoral cooperation (O) effectively

CMO 4 represents the refinement and consolidation of cCMO 4 and 5. The program adjustments emphasized in cCMO 4, and the consistency in procedures described in cCMO 5 have been synthesized into an adaptation mechanism employed in the refined cCMO 4. The focus of discussion in the FGD was on the recognition that PD/H, being a standardized intervention replicated in various community settings, requires differentiated approaches. Adaptation becomes a keyword in the mechanism of CMO 4. It is related to adaptation to the urban context (also identified in CMO 4 about sanitation management), target population criteria, and time frame. Furthermore, they also emphasized that the outcomes should extend beyond merely fostering sustainable behavioral change and encompass a broader focus on fostering multi-sectoral cooperation.

Positive Deviance/Hearth is a community empowerment program replicated with the same procedures and criteria across different countries. The FGD highlighted how this “one-size-fits-all” replication limited the program’s effectiveness. For example, the program’s criteria specified that children aged 6–36 months could participate. However, infants aged 6–8 months often slept during the program, making participation less effective.

*“The challenge was that infants aged 6*,* 7*,* 8 months often slept during the Pos Gizi session. So*,* they did not eat. Even when taken home*,* their mothers might not feed them” (Female*,* academia partnership Facilitator*,* March 2023).*

The program’s strict time frame, driven by the fiscal year of the funding NGO, also posed a significant challenge. The duration of the program was often too short to achieve the ambitious goal of improving child nutrition within a three-month intervention period.

*“But after cross-checking*,* it turns out that NGO’s standard of 900 grams for children with difficulty gaining weight is too high“(Female*,* academia partnership Facilitator*,* March 2023).*

With such high targets, program implementers were still required to work quickly within the organization’s time frame.

*“The implementation time is also very tight*,* so it becomes whatever it is. The important thing is to get it done” (Female*,* NGO Facilitator*,* March 2023).*

*“It should be that after training the volunteers*,* they should be given a month for a trial. But last time*,* it was immediate*,* and the time was tight” (Female*,* academia partnership Facilitator*,* March 2023).*

Intervention planning considers preparation for the intervention (including ensuring the time frame for strengthening implementers’ competencies) and determining the timing, monitoring, and evaluation methods. These conditions drive a redefinition of the expected outcomes, shifting the focus from behavior change to strengthening cross-sectoral cooperation. This perspective stems from the recognition that in the PD/H process, the partnership goes beyond academia-NGO partnerships.

#### CMO 5—Programs initiated by non-governmental organizations (C) require support in the form of commitment and involvement from multiple sectors, including the government office, (M) to build complex multi-sectoral cooperation (O)

A non-governmental organization initiated the Positive Deviance/Hearth program. However, it requires support from various sectors, including the government, the Public Health Center (Puskesmas), and government officials at the sub-district level.

*“I see that the Public Health Center (Puskesmas) is not yet fully committed to implementing the PDH program. So*,* the challenge of PDH is that it requires a lot of manpower. We know that even in Surabaya*,* the Puskesmas staff already have many tasks. They have their workload and salaries to deal with*,* let alone taking on additional work that is beyond their job descriptions” (Woman*,* NGO facilitator*,* March 2023).*

*“If an event or something was happening in the Sub-District*,* none of the Puskesmas staff came during the 10-day cooking demonstration. Hopefully*,* the Puskesmas will assist us. Because the mothers would be less trusting if only volunteers and cadres were involved. Having the Puskesmas staff present builds more trust” (Woman*,* student volunteer*,* March 2023).*

The Public Health Center (Puskesmas) plays a crucial role in supporting the implementation of a program in its jurisdiction. Government officials at the sub-district level contribute by providing community outreach and permitting Hearth sessions in their halls. Their support can come in the form of time and effort. Of course, this requires commitment, awareness, and cooperation between the organizers and the Puskesmas staff.

## Discussion

The five CMOs identified in this research illustrate how academia-NGO partnership can work on public health nutrition issues. We found that mechanisms involving various parties always come into play. Despite the involvement of academia and NGOs, this partnership cannot function effectively without the engagement of local governments and an understanding of the local context of the target population.

### PD/H intervention by non-governmental organizations in local urban communities

While CMOs 1 to 3 inform the importance of localizing the standardized international NGO intervention, all CMOs in our study indicate that collaborative programs between academia and NGOs should, at the very least, involve parents or caregivers, healthcare professionals, and sectors beyond healthcare.

The main challenges reported in implementing PD/Hearth relate to community participation and the consistency in reporting results that need improvement [7]. It is related to our first theory, which highlighted the involvement of parents during the program. Parental support, which commonly changed into caregiver support since most parents are working, includes participating in the Hearth sessions and practicing the meal menu at home after returning from the Hearth session. The cultural context in which the target communities reside has been identified as a key determinant of success and must be accommodated in implementing PD/Hearth, including in Indonesia [11]. Meanwhile, the implementation of this intervention in peri-urban areas in Cambodia indicates that the effectiveness of PD/Hearth requires supportive interventions to address mental health issues and the limitations of resources among caregivers of toddlers [12].

CMO 1 highlighted the importance of taking local context into account in interventions. Key characteristics of urban communities, such as the availability of time for caregivers, are critical to ensuring their involvement in the intervention. The Hearth session intervention carried out for 12 consecutive days in the morning during working hours is unsuitable for urban parents who are working. Additionally, CMO 1 indicated that interventions not addressing human-based design encourage caregiver motivation solely for financial rewards by participating in the intervention, resulting in negative impacts such as dependence on NGO assistance or donor incentives to attract community participation in public health programs [13]. PD/H requires the ability to accommodate these multiple interests of the target group to be able to foster their desire to adopt new health behaviors effectively [9]. When intervention does not consider the parties’ competing interests, it will not be easy to address the problem in real-world contexts [14].

PD/H intervention involves many implementers, ranging from NGOs to universities to health cadres. Since they have different backgrounds, there will be any potential to cause disharmony in understanding the intervention. CMO 2 shows that inequality occurs in skills, knowledge, commitment to procedures, and workload distribution. It leads to power imbalance and has repercussions on program operationalization at the implementation level [15]. CMO 2, which focuses on the standardization of implementer qualifications and workload, shows that the implementation plan should be able to define who will do what, how responsibilities are divided, and what is expected from each party. All parties involved in the partnership should be fully committed to participating and contributing effectively [16, 17]. This includes the provision of resources such as time and funds. Ensuring that the values and missions of both parties align is crucial [16, 18]. This will help build a strong foundation for sustainable partnerships.

The program’s sustainability is explained in CMO 3. To maintain the intervention’s ability in prevention and rehabilitation, the NGO-academia partnership needs to collaborate with other stakeholders, ensuring the children’s environment supports a healthy life. Public-private partnerships are often established between the government and Non-Governmental Organizations (NGOs) in the healthcare sector. However, without a shared platform between the government and NGOs in the intervention, the presence of NGOs is often perceived with unrealistic expectations from the community regarding healthcare services [19]. While NGO facilitation of maternal and child health programs run by the government can improve program equity, communities often perceive the presence of NGOs as providing free assistance without considering the program’s sustainability after the NGOs’ departure [20]. Collaborating with various parties to ensure proper sanitation management and preventive immunization will help form collective action and avoid dependence on NGOs’ financial support.

### Mechanisms underlying NGO collaboration with local universities

In the fourth CMO regarding program replication, there are indications that academia, as a collaborator, must adhere to the rules set by the NGO as the funder. This includes program time-framing that must align with the NGO’s schedule and program design that must follow the template provided by the NGO. In this research, participants never mentioned how the program was planned but consistently referred to it as a form of replication. Academia’s involvement in program planning only seemed evident during community mobilization and meal menu preparation.

Even though collaborating with the community offers the university a chance to prepare its students for real-world challenges and solutions, collaborating with academia has been documented as a partnership constrained by academic institutions’ time and human resources limitations [17]These time constraints are associated with the scheduling of faculty and students, which lacks significant flexibility due to conflicting class schedules and academic calendars. Additionally, concerns have arisen regarding the availability of personnel with the necessary training and expertise in specific competencies. This is because the primary actors responsible for engaging with the community are students in a learning phase who may not yet have sufficient experience.

Within the collaborations involving multiple organizations, distinct objectives, and performance metrics exist for each entity. These discrepancies and unclear role expectations and responsibilities undermine these partnerships’ quality [21]. The university expects that students can enhance the skills and knowledge they acquire in the classroom through practical work within the community [22]. At the same time, the partner and community expect that the program could run effectively under competent and well-trained facilitators [23, 24].

The environment and community needs can change over time. Therefore, it is important to have flexibility in this partnership and adapt to potential changes. Consider aspects of sustainability in this partnership, both financial and operational, to ensure that the program or project being implemented can be sustainable in the long run. Our research is conducted within a distinct local context, making it unique. While it addresses a topic commonly encountered in the field, the study’s findings may not universally apply. Instead, it serves as an exploratory investigation into a relatively unexplored subject.

## Conclusions

Using a case study of a partnership between the university and an NGO, this research highlights what makes their collaboration work and how it determines the outcomes. We found five context-mechanism-outcome configurations that made the PD/H intervention conducted under NGO-academia partnerships could be worked. Our findings illustrate the importance of considering the local context of the population when implementing standardized international NGO interventions. The involvement of various stakeholders besides academia and NGOs indicates that at the very least, collaborative programs between academia and NGOs should involve more stakeholders and build clearer expectations between stakeholders to minimize the dependency on NGOs and ensure the intervention’s sustainability. The community must show active participation, while the partnership must facilitate the participation. On the NGO side, the program must also be designed based on an equal power dynamic rather than the funder’s privilege.

## Supplementary Information

Supplementary Material 1.

Supplementary Material 2.

Supplementary Material 3.

## Data Availability

The data that support the findings of this study are available on request from the corresponding author. The data are not publicly available due to privacy or ethical restrictions.

## References

[1] Bermudez GF, Prah JJ. Examining power dynamics in global health governance using topic modeling and network analysis of Twitter data. BMJ Open. 2022;12(6): e054470. 35667718 10.1136/bmjopen-2021-054470 PMC9171232

[2] Charani E, Abimbola S, Pai M, Adeyi O, Mendelson M, Laxminarayan R, et al. Funders: the missing link in equitable global health research? Ventura DDFL, editor. PLOS Glob Public Health. 2022;2(6):e0000583. 36962429 10.1371/journal.pgph.0000583 PMC10021882

[3] Banerjee A. Policies for a Better-fed World (Working Paper No. 21623), Working Paper Series. National Bureau of Economic Research. 2015. 10.3386/w21623.

[4] UNICEF, WHO, World Bank Group. Levels and trends in child malnutrition: UNICEF/WHO/World Bank Group joint child malnutrition estimates: key findings of the 2023 edition. New York: UNICEF and WHO; 2023.

[5] UNICEF East Asia and the Pacific Regional Office. Southeast Asia regional report on maternal nutrition and complementary feeding. Bangkok: UNICEF; 2021.

[6] Gusnedi G, Nindrea RD, Purnakarya I, Umar HB, Susilowati A, et al. Risk factors associated with childhood stunting in Indonesia: a systematic review and meta-analysis. Asia Pac J Clin Nutr. 2023;32(2):184. 37382316 10.6133/apjcn.202306_32(2).0001

[7] Bisits Bullen PA. The positive deviance/hearth approach to reducing child malnutrition: systematic review: systematic review of the positive deviance/hearth approach. Tropi Med Int Health. 2011;16(11):1354–66. 10.1111/j.1365-3156.2011.02839.x 21749582

[8] Roche ML, Bury L, Yusadiredja IN, Asri EK, Purwanti TS, Kusyuniati S, et al. Adolescent girls’ nutrition and prevention of anaemia: a school based multisectoral collaboration in Indonesia. BMJ. 2018;363:k4541. 30530813 10.1136/bmj.k4541 PMC6282733

[9] Lapping K, Marsh DR, Rosenbaum J, Swedberg E, Sternin J, Sternin M, et al. The positive deviance approach: challenges and opportunities for the future. Food Nutr Bull. 2002;23(4 (supplement)):130. 12503241

[10] Chipili G, Chinyemba U, Ajayi K. The effect of positive deviance hearth approach on wasting among children aged 6–24 months in Chinkozya Community, Kazungula District, Southern Province Zambia. Indian J Nutri. 2021;8(3):235.

[11] Schooley J, Morales L. Learning from the community to improve maternal—child health and nutrition: the positive deviance/hearth approach. J Midwifery Womens Health. 2007;52(4):376–83. 17603960 10.1016/j.jmwh.2007.03.001

[12] Baik D, Reinsma K, Chhorvann C, Oy S, Heang H, Young MF. Program impact pathway of the positive deviance/hearth interactive voice calling program in a Peri-urban context of Cambodia. Curr Dev Nutr. 2022;6(5):6005011. 10.1093/cdn/nzac045 PMC9121803 35611354

[13] Shen C, Williamson JB. Child mortality, women’s status, economic dependency, and state strength: a cross-national study of less developed countries. Soc Forces. 1997;76(2):667–700.

[14] Duffy A, Christie GJ, Moreno S. The challenges toward real-world implementation of digital health design approaches: narrative review. JMIR Hum Factors. 2022;9(3): e35693. 36083628 10.2196/35693 PMC9508664

[15] Andress L, Hall T, Davis S, Levine J, Cripps K, Guinn D. Addressing power dynamics in community-engaged research partnerships. J Patient Rep Outcomes. 2020;4(1):24. 32249348 10.1186/s41687-020-00191-z PMC7131972

[16] Hushie M. Public-non-governmental organisation partnerships for health: an exploratory study with case studies from recent Ghanaian experience. 2016. 10.1186/s12889-016-3636-2 PMC5020518 27618964

[17] Mayer K, Braband B, Killen T. Exploring collaboration in a community-academic partnership. Public Health Nurs. 2017;34(6):541–6. 28762550 10.1111/phn.12346

[18] Gaihre S, Kyle J, Semple S, et al. Bridging barriers to advance multisector approaches to improve food security, nutrition and population health in Nepal: transdisciplinary perspectives. BMC Public Health. 2019;19:961 . 10.1186/s12889-019-7204-4. 10.1186/s12889-019-7204-4 PMC6637542 31319837

[19] Biermann O, Eckhardt M, Carlfjord S, Falk M, Forsberg BC. Collaboration between non-governmental organizations and public services in health – a qualitative case study from rural Ecuador. Globa Health Action. 2016;9(1): 32237. 10.3402/gha.v9.32237 PMC5112349 27852423

[20] Baqui AH, Rosecrans AM, Williams EK, Agrawal PK, Ahmed S, Darmstadt GL, et al. NGO facilitation of a government community-based maternal and neonatal health programme in rural India: improvements in equity. Health Policy Plann. 2008;23(4):234–43. 10.1093/heapol/czn012 18562458

[21] Liu M, Chung JE, Li J, Robinson B, Gonzalez F. A case study of community—academic partnership in improving the quality of life for asthmatic urban minority children in low-income households. IJERPH. 2022;19(15): 9147. 35897515 10.3390/ijerph19159147 PMC9332764

[22] Putri NK. Challenges in Public Health Program Planning exercise with a team-based learning approach during COVID-19 confinement. Indonesia J Med Educ. 2023;12(1):81.

[23] Putri NK, Prayoga D, Lailiyah S, Ernawaty E. Student-Community Partnership in Improving Health Literacy Toward National Health Insurance. Darmabakti Cendekia: Journal of Community Service and Engagements. 2021;2(2):70–75. 10.20473/dc.V2.I2.2020.70-75.

[24] Putri NK, Ridlo IA. How do health worker qualities affect collaborative skills? A qualitative study in Rural Indonesia Primary Health Services. Educ Med J. 2024;16(1):23–46. 10.21315/eimj2024.16.1.3.

