# Supplementary material for: Positive deviance/hearth intervention in collaboration between academia and NGOs: a realist evaluation
Source: BMC Public Health. 2024 Dec 28;24:3598. doi: 10.1186/s12889-024-20632-4 (PMC11681693; doi:10.1186/s12889-024-20632-4)
Supplement: Supplementary file 1 — Supplementary Material 1. [file 12889_2024_20632_MOESM1_ESM.docx]

**Appendix 1**

List of interviewees

| **Interviewee** | **Gender** | **Age (years)** | **Role in team** | **Base** |
| --- | --- | --- | --- | --- |
|  |  |  |  |  |
| Phase 1: Identifying program theories (In-depth interview) | | | | |
| I1 | Female | 25 | NGO program coordinators | NGO |
| I2 | Female | 24 | Academia coordinators | University |
| I5 | Female | 36 | Health cadre | Community |
| I6 | Female | 16 | Student Volunteer | University |
| Phase 2: Testing and refining program theories (Focus Group Discussion) | | | | |
| I1 | Female | 25 | NGO program coordinators | NGO |
| I2 | Female | 24 | Academia coordinators | University |
| I3 | Female | 46 | Health cadre | Community |
| I4 | Female | 22 | Student Volunteer | University |
| I5 | Female | 36 | Health cadre | Community |
| I6 | Female | 21 | Student Volunteer | University |
| I7 | Female | 40 | Health cadre | Community |
| I8 | Female | 23 | Student Volunteer | University |
| I9 | Female | 36 | Health worker | Public Health Center (Puskesmas) |
| I10 | Female | 30 | Health worker | Public Health Center (Puskesmas) |
