# Supplementary material for: Positive deviance/hearth intervention in collaboration between academia and NGOs: a realist evaluation
Source: BMC Public Health. 2024 Dec 28;24:3598. doi: 10.1186/s12889-024-20632-4 (PMC11681693; doi:10.1186/s12889-024-20632-4)
Supplement: Supplementary file 2 — Supplementary Material 2. [file 12889_2024_20632_MOESM2_ESM.docx]

**Appendix 2**

List of questions for interview and FGD

**Phase 1: Identifying program theories (In-depth interview)**

1. What are the initial objectives of establishing the PD/H program?
2. How those goals could be achieved? What has been and has not been achieved from all these goals? Why?
3. Are there other goals not previously set but discovered after performing PD/H? What is the purpose, and how is the process?
4. What factors influence success/failure in the implementation of PD/H? Why? What are the dominant factors that influence? Why?
5. What are the challenges and risks of implementing PD/H?
6. Is the implementation of the PDH in accordance with your expectations? Why?

**Phase 2: Testing and refining program theories (Focus Group Discussion**)

**Part 1**

The initial goal of the PDH program is firstly rapid rehabilitation of underweight and wasting children (Explanation of the definition of outcome one according to NGO guidelines).

"Quick rehabilitation for underweight and wasting children is provided by providing nutritionally dense foods during post-nutrition sessions which are held for ten days and provide two weeks for follow-up"

1. What context or resources support the achievement of this outcome? The context contains the resources that influence the ongoing process, infrastructure, information, and duration of implementation.

- What went well? (Asked after complete context)
- What needs to be improved?
- Who are the stakeholders whose involvement is needed to achieve these goals? How and what does it contribute to the program?

1. What processes support achieving these outcomes? Please provide an example

- What went well?
- What needs to be improved?

**Part 2**

The second goal is to prevent wasting and being underweight in the future. What do you think contributed to achieving this outcome?

"Because the underweight and wasting rate in each Surabaya sub-district is <30 or does not reach 30%, children with normal nutritional status are still included. "

Therefore, prevention involves monitoring children's growth with good nutritional status and ensuring they continue growing well.

- 1. What resources support achieving these outcomes? Please provide an example
- What went well? (asked after complete context)
- What needs to be improved?
- Who are the stakeholders whose involvement is needed to achieve these goals? How and what does it contribute to the program?

1. What processes support achieving these outcomes? Please provide an example

- What went well?
- What needs to be improved?

**Part 3**

The third goal is to maintain recovery results. What do you think contributed to achieving this outcome?

"Maintaining recovery results is done by carrying out home visits and also how to ensure the program can run sustainably"

- 1. What resources support achieving these outcomes? Please give examples
- What went well? (asked after complete context)
- What needs to be improved?
- Who are the stakeholders whose involvement is needed to achieve these goals? How and what does it contribute to the program?
  1. What processes support achieving these outcomes? Please provide an example
- What went well?
- What needs to be improved?

**Part 4**

Another goal that was not previously determined but was successfully found was a change in behavior by caregivers and children. What do you think contributed to achieving this outcome?

- 1. What resources support achieving these outcomes? Please give examples
- What went well? (asked after complete context)
- What needs to be improved?
- Who are the stakeholders whose involvement is needed to achieve these goals? How and what does it contribute to the program?
  1. What processes support achieving these outcomes? Please provide an example
- What went well?
- What needs to be improved?
