# Supplementary material for: Positive deviance/hearth intervention in collaboration between academia and NGOs: a realist evaluation
Source: BMC Public Health. 2024 Dec 28;24:3598. doi: 10.1186/s12889-024-20632-4 (PMC11681693; doi:10.1186/s12889-024-20632-4)
Supplement: Supplementary file 3 — Supplementary Material 3. [file 12889_2024_20632_MOESM3_ESM.docx]

**Appendix 3**

Qualitative Codebook

| Code | Definitions |
| --- | --- |
| **Context** | time, place, culture, and background that influence or give meaning to an event |
| **Mechanism** | the process or way in which something happens or is done |
| 1. Guardian | Parents or other relatives who take care of the children |
| - Working parent | parents who actively work, both in formal or informal jobs |
| - Commitment | willingness or dedication to comply with and participate in the program |
| - Involvement | willingness to donate time, energy, and other resources to support or participate in the program |
| 1. Implementer | NGO, university, or community |
| - Skills | the ability, knowledge, or expertise a person has in carrying out a task |
| - qualification | education, certification, work experience, or other criteria that determine whether a person is considered qualified or capable to perform a job |
| - workload | the number of tasks or responsibilities that a person or a team must complete in a certain period |
| - program guideline | standards, or steps that must be followed by those involved in the program |
| 1. Environment | the physical and social conditions that surround children |
| - Sanitation | practices or conditions related to environmental health and cleanliness |
| - Infection | a condition in which a child experiences an invasion of pathogenic organisms, which causes adverse biological reactions in their body due to an immune system that is not yet fully developed |
| **Outcome** | changes that occur as a result of PD/H interventions |
| - participation | Attendance of children and their guardians in each activity of PD/H |
| - prevention | A series of actions or steps taken to prevent wasting and underweight |
| - rehabilitation | recovering physical, mental, or social function due to wasting or being underweight |
| - Multi-sectoral cooperation | collaboration between various sectors or fields, both public, private, and civil society, to improve children's health |
